# Acute spontaneous intracerebral hemorrhage and traumatic brain injury are the most common causes of critical illness in the ICU and have high early mortality

**DOI:** 10.1186/s12883-018-1127-z

**Published:** 2018-08-27

**Authors:** Ye-Ting Zhou, Dao-Ming Tong, Shao-Dan Wang, Song Ye, Ben-Wen Xu, Chen-Xi Yang

**Affiliations:** 1 Department of Surgery, Affiliated Shuyang Hospital, Xuzhou Medical University, Xuzhou, Jiangsu China; 2 Department of Neurology, Affiliated Shuyang Hospital, Xuzhou Medical University, Xuzhou, Jiangsu China; 3 Department of Intensive Care Medicine, Affiliated Shuyang Hospital, Xuzhou Medical University, Xuzhou, Jiangsu China

**Keywords:** Critical illness, Causes, Incidence, Mortality, Epidemiology, Intracerebral hemorrhage, Head injury

## Abstract

**Background:**

Critical care covers multiple disciplines. However, the causes of critical illness in the ICU, particularly the most common causes, remain unclear. We aimed to investigate the incidence and the most common causes of critical illness and the corresponding early mortality rates in ICU patients.

**Methods:**

A retrospective cohort study was performed to examine critically ill patients (aged over 15 years) in the general ICU in Shuyang County in northern China (1/2014–12/2015). The incidences and causes of critical illnesses and their corresponding early mortality rates in the ICU were determined by an expert panel.

**Results:**

During the 2-year study period, 1,211,138 person-years (PY) and 1645 critically ill patients (mean age, 61.8 years) were documented. The median Glasgow Coma Scale (GCS) score was 6 (range, 3–15). The mean acute physiology and chronic health evaluation II (APACHE II) score was 21.2 ± 6.8. The median length of the ICU stay was 4 days (range, 1–29 days). The most common causes of critical illness in the ICU were spontaneous intracerebral hemorrhage (SICH) (26%, 17.6/100,000 PY) and traumatic brain injury (TBI) (16.8%, 11.4/100,000 PY). During the first 7 days in the ICU, SICH was the most common cause of death (42.2%, 7.4/10,000 PY), followed by TBI (36.6%, 4.2/100,000 PY). Based on a logistic analysis, older patients had a significantly higher risk of death from TBI (risk ratio [RR], 1.7; 95% CI, 1.034–2.635), heart failure/cardiovascular crisis (RR, 0.2; 95% CI, 0.083–0.484), cerebral infarction (RR, 0.15; 95% CI, 0.050–0.486), or respiratory failure (RR, 0.35; 95% CI, 0.185–0.784) than younger patients. However, the risk of death from SICH in the two groups was similar.

**Conclusions:**

The most common causes of critical illness in the ICU were SICH and TBI, and both critical illnesses showed a higher risk of death during the first 7 days in the ICU.

## Background

Twenty years ago, patients with organ failure accounted for most intensive care unit (ICU) cases [1], and a high mortality rate was observed in patients who refused ICU care [2, 3]. Today, with improvements in living standards and the aging of the general population, management of critically ill patients using intensive nursing care is more common [4–6]. Multiple epidemiological studies on prehospital emergency medical services have been conducted [7, 8]; however, the causes of critical illnesses in the ICU, especially the most common causes, remain unclear. Moreover, it is important to determine the most common illnesses that require management in an ICU and to understand the early mortality rates associated with these illnesses during the ICU stay. The aim of this study was to investigate the incidences and causes of critical illnesses and the corresponding early mortality rates among patients admitted to the ICU from the general population of Shuyang County in northern China.

## Methods

### Study design and participants

We retrospectively analyzed data that were collected from January 2014 to December 2015 for critically ill patients who were admitted to a general ICU (17 beds) in a tertiary teaching hospital—the unique three-general hospital. The hospital has a referral center (with 10 ambulances) and was responsible for all critical or emergency work within Shuyang County in northern China (38 county townships and a county town). Thus, 24 h a day, almost all critically ill patients were sent directly to the ICU. Therefore, this hospital was a strong source of critically ill patients, which ensured sufficient data availability. The study sample was compiled from the 2014 and 2015 data registries. During the study period, 1,211,138 people aged 15 years or older, including 697,615 women and 115,355 people aged 65 years or older, lived in the county [9]. The study was approved by the Ethical Committee on Clinical Research of the Shuyang People’s Hospital in China. Because the study involved care for critical illness, written informed consent was obtained from the nearest relative or a person who had been designated to provide consent by the patient.

### Procedures

To date, there are no compulsory diagnostic criteria for critical illness. Moreover, the codes for critical illnesses per the International Classification of Diseases, 10th Revision (ICD-10), are not well established. The relevant code changes according to the condition of the critically ill patient, and these conditions include coma (R40.2), cardiac arrest (I46.9), heart failure (I50), shock (R57.9), respiratory failure (J96), cardiovascular crisis and other critical events. We also identified those patients who had acute spontaneous intracerebral hemorrhage (SICH) by the code I61.9, but in this study, SICH was only limited to those with acutely non-traumatic coma. Similarly, traumatic brain injury (TBI) indicated a severe TBI (including traumatic subdural and epidural hematoma, or with intracerebral hematoma or subarachnoid hemorrhage) with sudden traumatic coma. Coma refers to a state of unconsciousness from which the patient cannot be aroused and is typified by the absence of language and motor function with a Glasgow Coma Scale (GCS) score of 3 to 8 (on a scale of 3 to 15, with lower scores indicating a lower level of consciousness). Cardiovascular crisis includes cardiogenic shock, cardiac arrest, sudden chest pain, and hypertensive crisis. Here, our retrospective study of ICU patients used the following inclusion criteria: (1) the patient (age > 15 years) exhibited an emergency- onset condition with unstable life signs or acute organ dysfunction and required transfer by emergency services to the ICU, and (2) the patient was hospitalized due to the onset a life-threatening organ dysfunction and required monitoring in the ICU. We used the following exclusion criteria: (1) age < 15 years, (2) an additional visit to the ICU within 2 weeks, and (3) residence in the county for < 6 months. We collected the critically ill patient data from the prospective registration of their primary diagnosis in the ICU patient out-of-hospital registry. Registration was the responsibility of the physician in charge of the ICU. The data registry annual volumes included sex, age, address, the date of admission, the sole primary diagnosis, the ICU stay length, the outcome of the main processing, and a contact telephone number for the ICU patient. All patients with craniocerebral disease were verified using their electronic medical records (an emergency cranial CT scan upon admission) and were assigned an initial GCS score and an acute physiology and chronic health evaluation II (APACHE II) score.

The time window of the ICU stay for most patients was between 3 and 7 days. Early mortality was assessed during the first 7 days of the critical illness. The World Health Organization (WHO) defines a person who is over 65 years old as geriatric. Therefore, patients were divided into a geriatric group and a non-geriatric group to compare the risks of critical illness between age groups.

Death events in the ICU and their causes were identified through medical record notes and from follow-up information. Follow-up information was from our physician who was conducted inquiries by phone to the patient’s closest living relative on day 7 of discharge from the ICU. Intensive care and neurology experts conducted a retrospective statistical analysis of the data of the study population during the 2-year period.

### Statistical analysis

All numeric variables are expressed as the mean ± SD or median (interquartile range [IQR]) and N (%). The overall incidence and causes were analyzed. The incidence rate per year in the population of critically ill patients was documented. Incidence rates for risk during 1 year were also calculated by dividing the baseline population by the number of annual cases. We used a logistic regression analysis to determine the risk ratio (RR) for a death event comparing the geriatric patients with the non-geriatric patients. All *p*-values were 2-sided, and significance was set at *p* < 0.05. Data were analyzed using SPSS version 17.0 (SPSS Inc., Chicago, IL, USA).

## Results

### Overall analysis

During the 2-year study period, 1791 critically ill cases were registered in the ICU following hospital discharge. We excluded cases that did not meet the inclusion criteria, including patients aged < 15 years (*n* = 126), patients who had another visitation to the ICU within 2 weeks (*n* = 21), and patients who had lived in the area for < 6 months (*n* = 4). Ultimately, 1645 patients who met the criteria were designated as critically ill patients and were enrolled into the study. The survey included 1015 men (61.7%) and 630 women (38.3%), which resulted in a male-to-female ratio of 1.7:1. The ages of the patients ranged from 15 to 98 years, and the average age was 61.8 ± 0.49 years. The median initial GCS score was 6 (range, 3–15). The mean initial APACHE II score (mean ± SD) was 21.2 ± 6.8. The median length of the ICU stay was 4 days (up to 29 days).

### Incidence of critical illness

During the 2-year study period, 1645 patients with an acute critical illness were documented among the total county population. The total incidence of critical illness was 67.9/100,000 person-years (PY) (Table 1). The most common acute critical illness types were strokes and neurological critical illnesses (632/2 years, 38.4%, 26.1/100,000 PY, including 577 cases of stroke/2 years, 35.1%, 23.8/100,000 PY), followed by trauma and traumatic brain injury (TBI) (397/2 years, 24.1%, 16.4/100,000 PY), cardiovascular critical diseases (140/2 years, 5.8/100,000 PY), and respiratory critical diseases (96/2 years, 4.0/100,000 PY).

**Table 1:** Incidence and ranking of emergency critical events

Critically ill events ranking	Number of cases in 2 years	Percentage (%)	Per100000 population members per year
Stroke and neurological critical ill	632	38.4	26.1
TBI and Trauma	397	24.1	16.4
Cardiovascular Critical ill	140	8.5	5.8
Respiratory Critical ill	96	5.8	4.0
After surgery or intervention	89	5.4	3.7
Gastrointestinal critical ill	74	4.5	3.1
Pesticides / drug poisoning	72	4.4	3.0
Sepsis / septic shock	48	2.9	2.0
Any cancer	32	1.9	1.3
Others	65	4.0	2.7
All	1645	100	67.9

*TBI* traumatic brain injury

The incidence of acute SICH tended to increase with increasing age, with the incidence peaking between 55 and 64 years. The incidence of TBI tended to decrease with decreasing age, with the incidence peaking between 65 and 74 years. The types and outcome of SICH and TBI during the first 7 days in the ICU are shown in Table 2.

**Table 2:** The types and outcome of SICH and TBI observed during the first 7 days in the ICU

Types	Number of cases in 2 years	Death (%)
SICH	427	180(42.2)
Striato-capsula,n(%)	178(41.7)	75(42.1)
Lobar,n(%)	81(10.0)	30(37.0)
Thalamus,n(%)	72(16.7)	23(31.9)
Brainstem, n(%)	61(14..3)	40(65.6)
Cerebellar, n(%)	26(6.1)	10(38.5)
Others (%),n(%)	9(2.1)	2(22.0)
TBI	276	101(36.6)
Subdural hematoma, n(%)	181(65.6)	89(49.2)
Intracerebral hemorrhage, n(%)	58(21.o)	9(15.5)
Epideural hematoma, n(%)	37(13.4)	3(8.1)
With subarachnoid hemorrhage, n(%)	201((72.8))	N/A
With contusions, n(%)	189(68.5)	N/A
With fractures, n(%)	182(65.9)	N/A

*ICU* intensive care unit, *SICH* spontaneous intracerebral hemorrhage, *TBI* traumatic brain injury

### Causes of critical illness

**Fig. 1:**
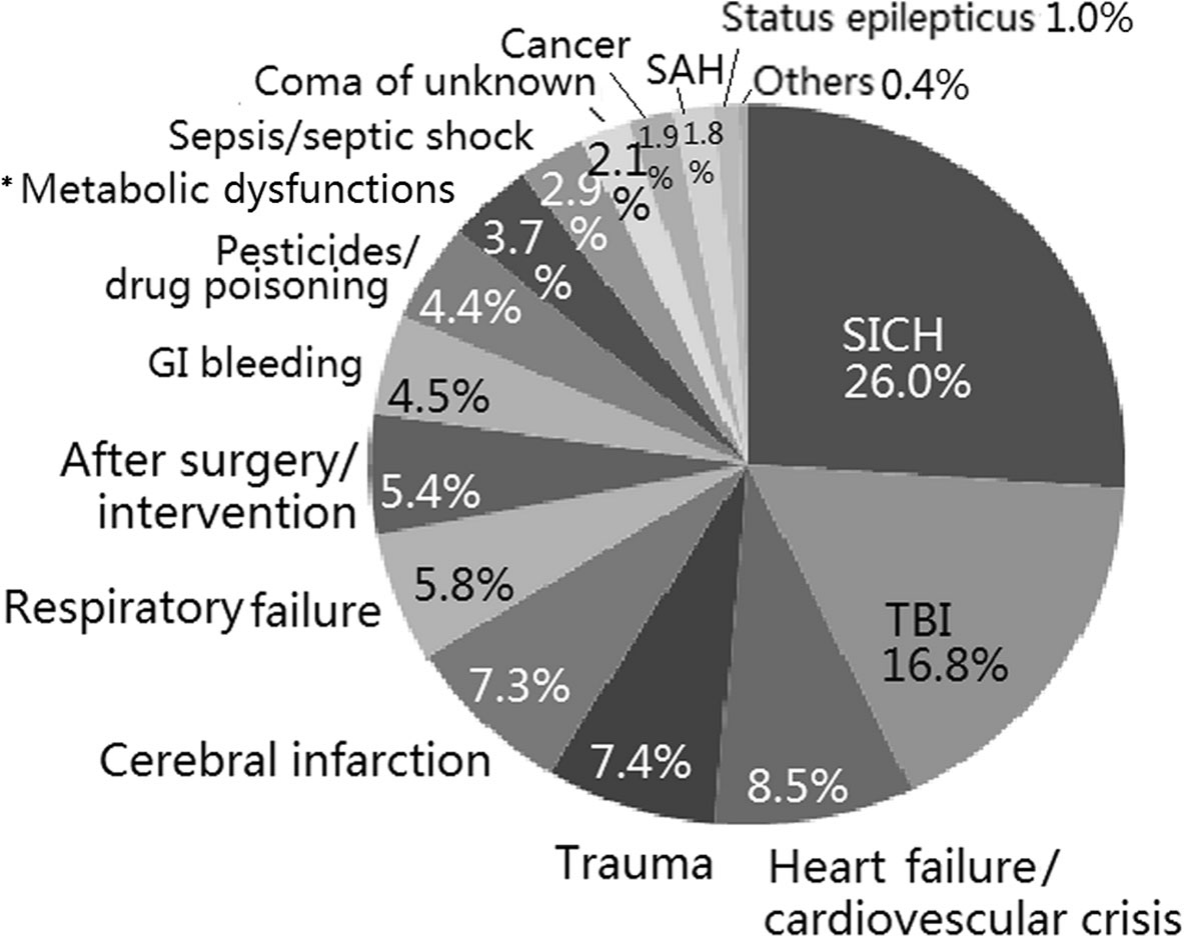
The causes of critical illness in a northern China ICU. *Metabolic dysfunctions: including hypoglycemia, diabetic ketoacidosis, hyperglycemic nonketotic hyperosmolar states, kedney failure or uremia, hepatic encephalopathy, heat stroke, anoxia, and electrolyte dysfunction, etc.

Of the 1645 cases of critical illness, the most common cause of critical illness was SICH with coma (26%, 17.6/100,000 PY), followed by acute TBI with coma (16.8%, 11.4/100,000 PY), heart failure/cardiovascular crisis (8.5%, 5.8/100,000 PY), trauma (7.4%, 5.0/100,000 PY), cerebral infarction with coma (7.3%, 5.0/100,000 PY), respiratory failure (5.8%, 4.0/100,000 PY), post-surgery complication or intervention (5.4%, 3.7/100,000 PY), pesticide/drug poisoning (4.4%, 3.0/100,000 PY), metabolic dysfunction (3.7%, 2.5/100,000 PY), and gastrointestinal bleeding with shock (3.4%, 2.3/100,000 PY). Other causes of critical illness were less frequent (Fig. 1).

### Incidence of death from critical illness

Four hundred eighty-nine deaths were reported in the ICU within the first 7 days of admission, with a rate of 20.2/100,000 PY. Males were at higher risk for this outcome than females (313/1015, 13.0/100,000 PY vs. 176/630, 7.4/100,000 PY, respectively; *p* < 0.005), and older patients (> 65 y old) were at higher risk than younger patents (15 y to 65 y old) (243/701, 10/100,000 PY vs. 246/944, 1/100,000 PY, respectively; *p* < 0.0001). (Table 3).

**Table 3:** Incidence of death due to critical illness during the first 7 days in the ICU

Charateristics	Number of cases in ICU/2 years	Death during the first 7 days in the ICU/2 years	Percentage (%)	Mortality during the first 7 days/per100000 population members per year
Overall	1645	489	29.7	20.2
Sex				
Male	1015	313	30.8§	12.9
Female	630	176	27.9§	7.3
Age				
> 65 years	701	243	34.7△	10.0
15 to 65 years	994	246	24.7△	1.0
Types of critical illness				
SICH	427	180	42.2	7.4
TBI	276	101	36.6	4.2
Heart failure/cardiovescular	140	61	43.6	2.5
Respiratory failure crisis	96	28	29.2	1.2
After surgery or intervention	89	10	11.2	0.4
GI bleedingwith shock	74	22	29.7	0.9
Cerebral infarction	120	21	17.5	0.9
Sepsis / septic shock	48	21	43.8	0.9
Pesticides/ drug poisoning	73	11	15.1	0.5
Coma of unknown origin	35	9	25.7	0.4
Trauma	121	8	6.6	0.3
Any cancer	32	7	21.9	0.3
SAH	30	7	23.3	0.3
Metabolic dysfunction	61	2	3.3	0.1
Status epilepticus	16	1	6.2	0.04
Other	7	0	0	0.0

§*p* < 0.005; △*p* < 0.0001

*ICU* intensive care unit, *SICH* spontaneous intracerebral hemorrhage, *TBI* traumatic brain injury

### Causes of death from critical illness in the ICU

The causes of death from critical illness within the first 7 days of admission to the ICU were determined. The most common cause of death for ICU patients was SICH (180 patients, 7.4/100,000 PY), followed by acute TBI (4.2/100,000 PY), heart failure/cardiovascular crisis (2.5/100,000 PY), respiratory failure (1.2/100,000 PY), cerebral infarction (0.9/100,000 PY), gastrointestinal bleeding (0.9/100,000 PY), sepsis/septic shock (0.9/100,000 PY), coma of unknown origin (0.4/100,000 PY), pesticide poisoning (0.5/100,000 PY), other traumas (0.3/100,000 PY), cancer (0.3/100,000 PY), SAH (0.3/100,000 PY), metabolic dysfunction (0.1/100,000 PY), and status epilepticus (0.04/100,000 PY) (Table 3).

### Risk of death within 7 days of admission for the geriatric group vs. the non-geriatric group

Using a logistic regression analysis of the same study population, we compared the risk of death within 7 days of admission between the geriatric group and the non-geriatric group (Table 4). The risks of death from TBI (RR, 1.7; 95% CI, 1.034–2.635; *p* < 0.05), cerebral infarction (RR, 0.15; 95% CI, 0.050–0.486; *p* < 0.0005), heart failure/cardiovascular crisis (RR, 0.2; 95% CI, 0.083–0.484; *p* < 0.0005), and respiratory failure (RR, 0.35; 95% CI, 0.185–0.784; *p* < 0.005) were higher in the geriatric group than in the non-geriatric group. The risks of death from SICH, cardiac arrest with resuscitation, gastrointestinal bleeding, sepsis/septic shock, pesticide poisoning, and coma of unknown origin were not significantly different between the two age groups.

**Table 4:** Logistic regression analysis of the risk of death comparing the geriatric group and non-geriatric group during the first 7 days in the ICU

Critical illness	RR	95%CI	*p* Value
TBI	1.7	1.034–2.635	0.034
Heart failure/cardiovascular crisis	0.2	0.082–0.483	0.000
Cerebral infarction	0.15	0.049–0.460	0.001
Respiratory failure	0.35	0.159–0.790	0.011

*ICU* intensive care unit, *RR* risk ratio, *CI* confidence intervals, *TBI* traumatic brain injury

## Discussion

In previous epidemiological surveys of prehospital emergency care, altered mental status [7] and trauma [8] have been reported to have the highest incidence rates. However, this study found that the incidences of these ailments among critically ill patients in the ICU were different from those observed in previous prehospital emergency care studies. We found that stroke (35.1%, 23.8/100,000 PY) had the highest incidence rate, followed by trauma and TBI (24.1%,16.4/100,000 PY). The latter incidence may be due to the number of mild condition patients with prehospital trauma and TBI that may have been managed outside the ICU. This has been confirmed by a recent study that reported that only 20% of TBI cases account for admission to the ICU [10]. Conversely, epidemiological studies have confirmed that the incidence [11] and mortality rate for stroke are very high in China [12]. Therefore, it is likely that the care of patients with stroke has become a primary objective of ICU management.

The incidence of SICH varies in countries around the world [13], but recent studies have confirmed that the SICH incidence rate is highest in Asians (40.8–51.8/100,000 PY) [13, 14]. According to our study, SICH is a main cause of critical illness among patients admitted to the ICU, which suggests that more than 1/3 of patients with SICH need ICU care. Moreover, the fatality rate for patients with SICH within the first 7 days of admission was very high (42.2%) and similar to the fatality rate after 1 month (43–52%) that has been reported by other studies [15, 16].

TBI is the primary cause of injury-related death and disability [17], although its exact incidence is unclear. Our current study showed that TBI (16.8%, 11.4/100,000 PY) was the second most common cause of ICU admission. TBI has a mortality rate of approximately 40.0% [18] and a disability rate of 57.4% [19]. Moreover, our data showed that the fatality rate within the first 7 days of admission was also high in cases of TBI (36.6%). However, the risks of death from TBI, heart failure/myocardial infarction, severe cerebral infarction, and respiratory failure were significantly higher in the geriatric group than in the non-geriatric group. This finding indicates that older age is an important risk factor for death from most critical illnesses in the general ICU, which is consistent with a previous study [20] and may be related to that the older patients is more likely to develop an acutely fatal multiple organ failure [21]. On the contrary, the risk of death from SICH in the two groups was similar, and the SICH incidence results suggested a trend toward an increase in the younger population. This has been confirmed by one recent study [22].

Prior studies have shown that sepsis and septic shock account for 10% of ICU cases [23], whereas this study identified only 48 such cases (2.9%). Because most sepsis or septic shock diagnoses are secondary to another disease, our result may be related to our study criteria, which examined only the first diagnosis (primary disease). These patients had been transferred to specialist wards or information on sepsis/septic shock had not been included in the diagnosis.

Surprisingly, the combined incidence of SICH and TBI was almost equal to the sum of the general ICU admissions for other critical patients. This strongly suggests that addressing these two critical diseases should be a public health priority and is deserving of further attention.

Although this study was conducted in one ICU, this ICU was the county’s only ICU with a referral center. Therefore, we believe that our data are informative because the results of this study are more likely to represent the true epidemic spectrum of critical illness. However, certain limitations should be acknowledged. First, although this was a prospective registration, a small number of critically ill patients may be underrepresented due to a lack of standardized criteria for defining critical illness in this population. Second, because our registration only selected a sole primary diagnosis in the ICU, some patients with sequential liver or renal failure were not documented. This may have been the cause of the low frequencies of liver and renal failures that we observed. Additionally, the causes of coma were difficult to identify because numerous comatose patients had been intubated and could not consent to brain magnetic resonance examination. However, patients with unexplained comas were rare, so this was unlikely to produce a bias in the population of critically ill patients with neurological conditions.

In conclusion, the rate of critical illness was high in Shuyang County in northern China. The most common illnesses that required care in the ICU were SICH and TBI, and both of these critical illnesses carried a higher risk of death than other illnesses. This strongly suggests that addressing these two conditions should be a public health priority and is deserving of further attention.

## Abbreviations

APACHE II: Acute physiology and chronic health evaluation II
GCS: Glasgow coma scale
ICU: Intensive care unit
SICH: Spontaneous intracerebral hemorrhage
TBI: Traumatic brain injury
